# Molecular Detection, Phylogenetic Analysis, and Identification of Transcription Motifs in Feline Leukemia Virus from Naturally Infected Cats in Malaysia

**DOI:** 10.1155/2014/760961

**Published:** 2014-11-17

**Authors:** Faruku Bande, Siti Suri Arshad, Latiffah Hassan, Zunita Zakaria

**Affiliations:** ^1^Department of Veterinary Pathology and Microbiology, Faculty of Veterinary Medicine, Universiti Putra Malaysia, 43400 UPM Serdang, Selangor, Malaysia; ^2^Department of Veterinary Services, Ministry of Animal Health and Fisheries Development, PMB 2109, Usman Faruk Secretariat, 840221 Sokoto, Sokoto State, Nigeria; ^3^Department of Veterinary Laboratory Diagnostics, Faculty of Veterinary Medicine, Universiti Putra Malaysia, 43400 UPM Serdang, Selangor, Malaysia

## Abstract

A nested PCR assay was used to determine the viral RNA and proviral DNA status of naturally infected cats. Selected samples that were FeLV-positive by PCR were subjected to sequencing, phylogenetic analysis, and motifs search. Of the 39 samples that were positive for FeLV p27 antigen, 87.2% (34/39) were confirmed positive with nested PCR. FeLV proviral DNA was detected in 38 (97.3%) of p27-antigen negative samples. Malaysian FeLV isolates are found to be highly similar with a homology of 91% to 100%. Phylogenetic analysis revealed that Malaysian FeLV isolates divided into two clusters, with a majority (86.2%) sharing similarity with FeLV-K01803 and fewer isolates (13.8%) with FeLV-GM1 strain. Different enhancer motifs including NF-GMa, Krox-20/WT1I-del2, BAF1, AP-2, TBP, TFIIF-beta, TRF, and TFIID are found to occur either in single, duplicate, triplicate, or sets of 5 in different positions within the U3-LTR-gag region. The present result confirms the occurrence of FeLV viral RNA and provirus DNA in naturally infected cats. Malaysian FeLV isolates are highly similar, and a majority of them are closely related to a UK isolate. This study provides the first molecular based information on FeLV in Malaysia. Additionally, different enhancer motifs likely associated with FeLV related pathogenesis have been identified.

## 1. Introduction

Feline leukaemia virus (FeLV) is a gammaretrovirus associated with anaemia, immunodeficiency, leukaemia, and lymphoma in cats [1]. FeLV has been studied extensively as a model for human immunodeficiency virus (HIV) and human T-cell lymphoma virus (HTLV) infections [2]. FeLV is distributed worldwide; however, prevalence varies greatly with geography and with risk factors that include age, health status, and population density [3, 4]. A recent study reported FeLV seroprevalence of 5.1% and 18.9% in healthy and sick Malaysian cats, respectively [4]. On the other hand, studies carried out in other Asian regions reported 0% FeLV prevalence in Vietnam [5]; 14.7% among cats in Singapore [6]; 2.9% in Japan [7]; and 6% each from Taiwan and Thailand [8, 9]. In non-Asian countries, FeLV prevalence has been reported to be 4.8% on Prince Edward Island, Canada [10]; 5.3% and 3.7% in Raleigh and Gainesville, USA, respectively [11]; 3.4% in all Canada [12]; and 4.6% in Egypt [13]. These discrepancies in prevalence of FeLV may arise due to differences in cat's lifestyle and FeLV vaccination practices in different countries [4].

Diagnosis of FeLV is usually performed by detection of p27 antigen [14]. However, demonstrating p27 antigen is difficult during early viraemia and with latent infections. Studies have shown that FeLV viral RNA and provirus DNA are better predictors of progressive and latent infections, respectively [15, 16].

Apart from the envelope gene of FeLV, the long terminal repeats (LTRs) play important role in determining disease outcome and in differentiating exogenous from endogenous FeLV [15, 17]. Vaccination against FeLV is not carried out in Malaysia and, to date, FeLV clinical status of Malaysian cats has not been investigated using molecular assays. Additionally, unlike the ubiquitous feline infectious peritonitis (FIP) [18] sequence and phylogenetic characteristics of the Malaysian FeLV isolates have not been elucidated. The objectives of this study are to evaluate the presence of FeLV viral RNA and provirus DNA in selected antigenaemic and nonantigenaemic cats, respectively. Sequence characteristics, enhancer motifs as well as phylogenetic relationships of the Malaysian FeLV also were determined.

## 2. Materials and Methods

### 2.1. Animals and Sampling

Heparinized blood samples were collected from cats presented at University Veterinary Teaching Hospital, Universiti Putra Malaysia (UVH-UPM). The samples were tested for the presence of FeLV p27 viral antigen using a commercially available test kit [4]. These cats were divided into p27 antigen positive and p27 antigen-negative groups. From each group, 39 cats were selected by convenience sampling method and the samples were subjected to PCR analysis. All cats had no history of vaccination against FeLV as vaccination against FeLV is not practiced in Malaysia. All samples were collected by the attending veterinary clinicians, as part of routine practices. In addition, consent for evaluation was obtained from the cat owners, prior to sampling.

### 2.2. Nucleic Acid and PCR Amplification

Viral RNA was extracted from the plasma of p27-positive cats, using high pure viral RNA purification kit (Roche, Germany). On the other hand, genomic DNA was isolated from whole blood of p27-negative cats, using QIAGEN DNA extraction kits (QIAGEN, Germany). All nucleic acid extraction procedures were carried out according to manufacturers' instructions. RNA was reverse transcribed and subjected to nested PCR, using a one-step access RT PCR (Promega, USA). Genomic DNA was amplified by nested PCR assay.

Two sets of primers (outer and inner primers) were synthesised (1st BASE, Malaysia) and used to amplify a 601 bp segment of FeLV-U3LTR and partial* gag* regions. This segment recognises exogenous but not endogenous FeLV segments presence in cat genome; thus the primers used in this study are specific for exogenous FeLV detection. Outer PCR reaction was carried out using U3-F(1) (5′-ACA GCA GAA GTT TCA AGG CC -3′) and G-R(1) (5′-GAC CAG TGA TCA AGG GTG AG-3′) primers. The inner PCR reaction was carried out with U3-F(2) (5′-GCT CCC CAG TTG ACC AGA GT-3′) and G-R(2) (5′-GCT TCG GTA CCA AAC CGA AA-3′) primers [15].

The PCR mixture was prepared in 25 *µ*L reaction volume containing 10 mM each of dNTPs mix, 0.2 mM* Tfl* DNA polymerase (5 U/*µ*L), 0.1 U AMV (5 U/*µ*L), 0.1 U recombinant RNasin ribonuclease inhibitor (400 U/*µ*L), 0.8 U MgSO_4_ (25 mM), 20 pmol of each of the forward and reverse primer, 5.0 *µ*L of 1 times buffer, and 1 *µ*L RNA or DNA template. Nuclease-free water was used to bring the mixture to its final volume of 25 *µ*L. AMV reverse transcriptase enzymes and RNasin ribonuclease inhibitor were included only when RNA was a starting template for the PCR assay. In the nested PCR step, 1 *μ*L of outer PCR product was used as template.

In both inner and outer PCR steps, the target gene regions were amplified using the following conditions: reverse transcription: 45°C (45 min) (only in the case of RNA), initial denaturation: 94°C (2 min), denaturation: 94°C (45 sec), annealing: 58°C (30 sec), extension: 72°C (1 min), 35 cycles of repeats, and final extension: 72°C (7 min). PCR product was electrophoresed using 1.5% agarose (SeaKem LE USA), stained with 0.5 *µ*g/mL ethidium bromide (Bio-Rad USA), and visualised under UV light (Geldoc system, Bio-Rad, USA). Extraction and amplification procedures were carried out in separate hood to reduce chances of contamination.

### 2.3. Sequence and Phylogenetic Analyses

In order to gain insight on the characteristics of Malaysian FeLV sequences, 29 nested PCR-positive samples (RNA *n* = 14; DNA provirus *n* = 15) were selected and purified using an Accuprep purification kit (Bioneer, Daejeon, Korea). Sequencing was carried out based on the amplified U3LTR-gag segment using a standard ABI Big Dye terminator version 3.1 sequence kit (Applied Biosystem). The obtained sequences were analysed for homology using the NCBI Basic Local Alignment Search Tool (BLAST: http://www.ncbi.nlm.nih.gov). In addition, multiple sequence alignment was carried out using ClustalW and the percentage nucleotide identity was determined using DNA identity matrix [19, 20]. On the other hand, single nucleotide polymorphism (SNP), DNA distance matrix, and transcription binding proteins prediction analyses were carried out using geneious software version R7 [20]. A neighbour-joining (NJ) phylogenetic tree was constructed based on the U3LTR-gag sequences using MEGA5 software. The tree reliability was assessed using 100 bootstrap replicates [21]. All nucleotide sequences were deposited with the NCBI GenBank (Table 1).

## 3. Results and Discussion

**Figure 1:**
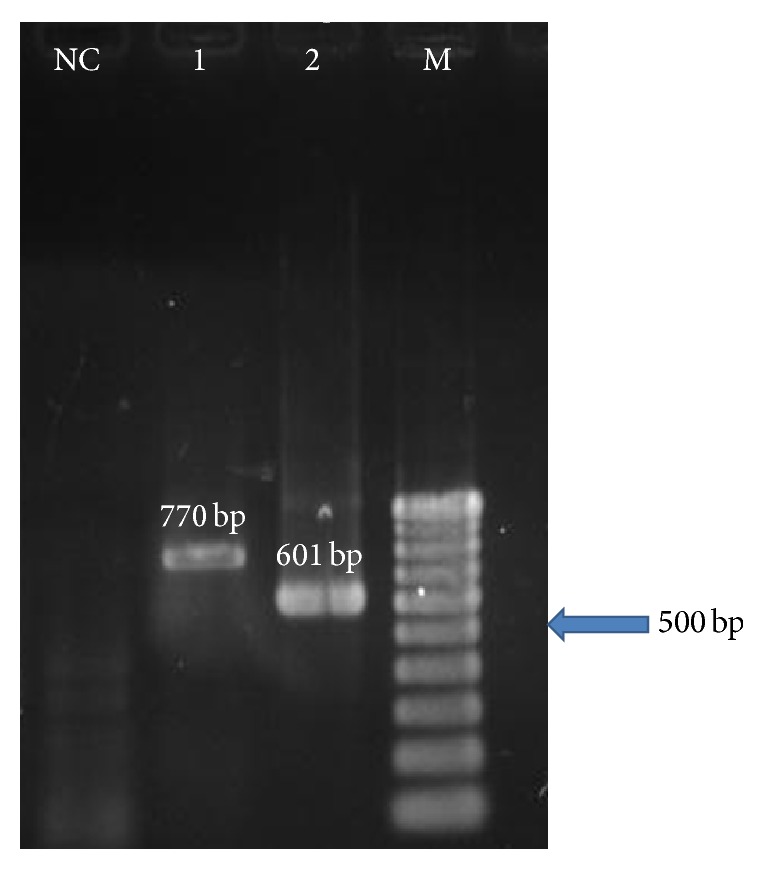
Nested RT-PCR assay used to detect FeLV U3LTR-gag sequence from naturally infected cats in Malaysia. The outer PCR reaction was performed using primer pair U3-F(1) and G-R(1) and amplified 770 bp (lane 1) whilst inner PCR reaction was performed using primer pair U3-F(2) and G-R(2) and amplified 601 bp (lane 2). Lane NC: negative control, lane M: 100 bp DNA marker (iDNA, Malaysia).

FeLV infection is of concern to cat owners due to its ability to induce tumours and immunodeficiency, thus predisposing cats to other secondary diseases. In this study, a U3-LTR and gag regions of exogenous without endogenous FeLV sequences were amplified by nested PCR methods. Post-PCR analysis using electrophoresis revealed an expected amplicon size of 770 bp in the outer PCR and 601 bp in the nested inner PCR assay (Figure 1). Overall, it was found that 97.4% (38/39) of p27 antigen-negative cats were positive for FeLV provirus DNA suggesting that this category of cats likely goes undetected when only p27 detection is used to judge their FeLV clinical status. Similar studies reported high prevalence of FeLV provirus DNA in Brazilian cats [22]. However, Hofmann-Lehmann et al. [23] reported a lower provirus DNA rate in cats in Switzerland. The observed differences in prevalence among different countries could be associated with cat lifestyle, as well as variations in factors known to favour FeLV transmission [3, 4]. Provirus DNA detection rate observed in this study could be associated with regressive or latent FeLV infection, which is characterized by integration of DNA provirus into the host cell genome and absence of viral antigen in circulation [1, 15].

The consequence of latent FeLV infection is that provirus DNA could reactivate to an infectious state, especially following stress and/or immunosuppression. Thus, cats that are p27 antigen-negative, but provirus DNA positive, could serve as sources of infection of FeLV-naïve cats [24]. A previous study has established an association between feline lymphoma and provirus DNA positivity in p27 antigen-negative cats, though this has not been evaluated in the present study [25]. Moreover, transmission of FeLV has been shown to occur in cats following blood transfusion from cats with provirus DNA, thus highlighting the importance of screening blood donor cats for provirus DNA [26].

Viral RNA was detected in 87.2% (34/39) of p27 antigen-positive cats whereas 13% (5/39) tested negative using RT-PCR assay. Since plasma viral RNA is an indicator of FeLV viraemia, cats that are positive for FeLV p27 antigen and viral RNA are likely to harbour replicating virus [27]. Cats in this category may progress to a persistent viraemic stage, succumbing to FeLV-associated illness [28].

Failure to detect FeLV viral RNA in about 13% p27 antigen-positive cats (p27-positive/viral RNA-negative) could result from atypical infection, wherein the virus is sequestered and replicates locally in tissues such as salivary gland, mammary gland, and urinary epithelium, causing intermittent or low-grade antigenaemia, although there is no detectable viraemia [28, 29]. Our findings are consistent with the results of an earlier study that failed to isolate FeLV from about 10% of p27 antigen-positive cats, irrespective of the antigen detection methods used. Such cats were considered as “discordant,” suggesting that p27 antigen-positive status may not always correlate with viraemia [30]. Another potential explanation for p27-positive-RNA-negative status might be false positive antigen or false negative RNA tests that arise occasionally because of low positive predictive value of p27 antigen tests in regions with low FeLV prevalence [31]. Clinical relevance of atypical FeLV infection is not well-understood, and it has been recommended to monitor the status of discordant cats over time [27, 28]. No additional follow-up was carried out in the present study, because most owners were not willing to subject cats to repeated venepunctures [4].

Based on the U3LTR and partial* gag* regions, nucleotide sequence analyses revealed homology of 91–100% among Malaysian FeLV isolates. However, homology decreased to 84.6% when local isolates were compared with reference isolates. Previous studies reported strong sequence conservation (>97%) among FeLV isolates of different geographic and temporal clusters [32, 33]. In agreement with Jackson et al. [17], we do observe point mutations and nucleotide deletion in Malaysian FeLV isolates (see Supplementary Material available online at http://dx.doi.org/10.1155/2014/760961).While U3LTR is conserved in FeLV, field isolates have been reported to exhibit sequence variation within the terminally repeated LTRs regions [17, 33]. Mutational changes in the LTR regions have been implicated with enhanced transcriptional and/or insertion activities of FeLV, thus supporting T-cell lymphomagenesis [34, 35].

In this study, several transcription binding motifs were predicted within the amplified U3LTR-gag region (Table 2). Of these, NF-GMa, Krox-20/WT1I-del2, BAF1, AP-2, TBP, TFIIF-beta, TRF, and TFIID motifs were found to be conserved between local FeLV isolates and the two characterized FeLV-Rickard subgroup A and FeLV-FAIDS reference isolates. On the other hand, E1A-F, ELP, Sp1, C∖EBPbeta, BAF1, GCF, HNF-3, and PEA3 motifs are found in some local isolates but were absent in reference sequences. These motifs may have implication for viral oncogenicity or probably favours viral replication. For example, an Sp1 enhancer, a member of Sp/Kruppel-like factor, was reported to activate gene transcription and contribute to abnormal metabolism of cancer cells [36, 37] whereas C∖EBPbeta regulates the growth and differentiation of myeloid as well as lymphoid cells [38]. Studies have shown that, the U3-LTR sequence contains multiple transcription binding sites that aid viral replication and pathogenesis. Interactions of different transcription binding factors, via the U3-LTRs, may contribute to cellular gene transactivation and viral leukemogenesis [39, 40]. Enhancer motifs observed in this study appeared in multiple locations such as in the case of E1A-F, BAF1, and TFIID, each occurring in duplicate; GCF appeared in triplicate while AP-2 is repeated 5 times at different positions. An enhancer duplication and triplication has been reported in naturally occurring cases of FeLV-induced T-cell lymphomas [41, 42]. The clinical relevance of multiple enhancers in cats used in the present study is not determined, although some FeLV positive cats had evidence of different tumour forms at post-mortem (result not shown). Previous studies reported that E1A-F, a member* ets*-oncogene family transcription factor, upregulates the multiple matrix metalloproteinase (MMP) genes thus contributing to the malignant phenotypic activity by increasing the invasion and metastatic activities of cancerous cells [43]. TFIID, a potential protooncogene with TATA-box protein and a TBP-associated factor also plays role in transcription initiation and genome expression [44]. On the other hand, AP2 and SP1 are known to activate epidermal growth factor receptor (EGFR) gene. In addition, overexpression of these gene has been reported to cause cellular transformation [45, 46]. Surprisingly we also identified a triplicate of GCF binding factor that has suppressor effect on EGR gene; these discrepancies, however, need further elucidation with quantitative real-time PCR [47].

Absence of length mutation (nucleotide position 473–481) in Malaysian FeLV isolates, as observed in FeLV isolates from Taiwan (FeLV-TW-25 and FeLV-TW-30) and a European isolate (FeLV-GM1), might suggest limited influence of geography in evolutionary patterns of FeLV, unlike its lentiviral counterpart, feline immunodeficiency virus [33, 48].

**Figure 2:**
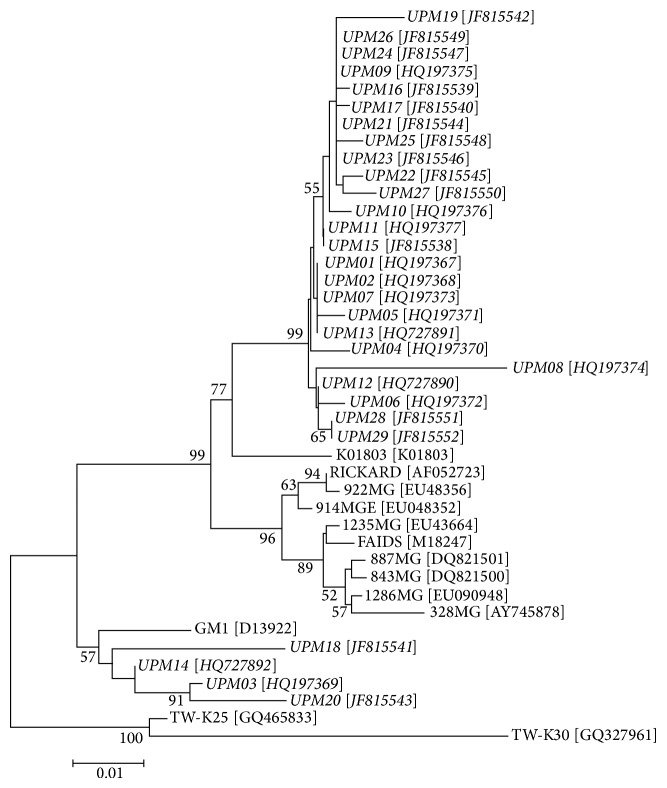
Neighbour-joining phylogenetic relationship of Malaysian FeLV isolates. The percentage of replicate trees in which the associated taxa clustered together in the bootstrap test (100 replicates) is shown next to the branches. Malaysian FeLV isolates are indicated in italic. Evolutionary analyses were conducted in MEGA5.

Phylogenetic analysis based on the U3LTR-gag sequence revealed that Malaysian FeLV isolates are closely related (Tables 3(a), 3(b), and 3(c)) but when compared with reference isolates, separated into two distinct clusters, with the majority (86.2%) being closely related to FeLV-K01803 isolate from UK. The remaining local FeLV isolates (13.8%) clustered with FeLV-GM1 (Figure 2). The reason for the observed similarity between local FeLV isolates and European isolates, but not with Taiwanese isolates, may suggest the lack of geographical influence, this should be explored further. It is possible also that FeLV might have been introduced into Malaysia as a result of translocation of domestic pets from Europe. Due to a somewhat conserved nature of the U3LTR region, conclusion about the FeLV subgroup requires further investigations of FeLV envelope protein gene.

## 4. Conclusion

This study revealed the occurrence of FeLV viral RNA and provirus DNA among naturally infected Malaysian cats. Based on the U3LTR-gag sequence, Malaysian FeLV isolates are highly conserved and more closely related to K01803 isolate from UK compared to Taiwanese and other reference isolates. Presence of multiple enhancers some of which have been linked with FeLV induced tumours may contribute to the development of poor prognostic outcome in naturally infected Malaysian cats although this needs further investigation. Overall, this is the first molecular study for evidence of FeLV in Malaysia. We also identified several motifs that have potential implications in FeLV-induced leukemogenesis. Future studies need to explore association between FeLV positive status and occurrence of feline tumour in Malaysian cats. The present findings is useful in designing molecular diagnostics for clinical applications and for improved understanding of FeLV infection outcome and epidemiology.

## Supplementary Material

Multiple sequence alignment of Malaysian FeLV with reference isolates.

## Figures and Tables

**Table 1:** List of local and reference sequences used in phylogenetic analysis of Malaysian FeLV isolates.

Isolate	Accession number	Country	Source
FeLV-UPM01	HQ197367	Malaysia	This study
FeLV-UPM02	HQ197368	Malaysia	This study
FeLV-UPM03	HQ197369	Malaysia	This study
FeLV-UPM04	HQ197370	Malaysia	This study
FeLV-UPM05	HQ197371	Malaysia	This study
FeLV-UPM06	HQ197372	Malaysia	This study
FeLV-UPM07	HQ197373	Malaysia	This study
FeLV-UPM08	HQ197374	Malaysia	This study
FeLV-UPM09	HQ197375	Malaysia	This study
FeLV-UPM10	HQ197376	Malaysia	This study
FeLV-UPM11	HQ197377	Malaysia	This study
FeLV-UPM12	HQ727890	Malaysia	This study
FeLV-UPM13	HQ727891	Malaysia	This study
FeLV-UPM14	HQ727892	Malaysia	This study
FeLV-UPM15	JF815538	Malaysia	This study
FeLV-UPM16	JF815539	Malaysia	This study
FeLV-UPM17	JF815540	Malaysia	This study
FeLV-UPM18	JF815541	Malaysia	This study
FeLV-UPM19	JF815542	Malaysia	This study
FeLV-UPM20	JF815543	Malaysia	This study
FeLV-UPM21	JF815544	Malaysia	This study
FeLV-UPM22	JF815545	Malaysia	This study
FeLV-UPM23	JF815546	Malaysia	This study
FeLV-UPM24	JF815547	Malaysia	This study
FeLV-UPM25	JF815548	Malaysia	This study
FeLV-UPM26	JF815549	Malaysia	This study
FeLV-UPM27	JF815550	Malaysia	This study
FeLV-UPM28	JF815551	Malaysia	This study
FeLV-UPM29	JF815552	Malaysia	This study
FeLV-914MG	EU048352	Brazil	GenBank
FeLV-1235MG	EU43664	Brazil	GenBank
FeLV-843MG	DQ821500	Brazil	GenBank
FeLV-887MG	DQ821501	Brazil	GenBank
FeLV-922MG	EU48356	Brazil	GenBank
FeLV-1286MG	EU090948	Brazil	GenBank
FeLV-328MG	AY745878	Brazil	GenBank
FeLV-Rickard	AF052723	USA	GenBank
FeLV-FAIDS	M18247	USA	GenBank
FeLV-GM1	D13922	UK	GenBank
FeLV-K01803	K01803	UK	GenBank
FeLV-TWK25	GQ465833	Taiwan	GenBank
FeLV-TW-K30	GQ327961	Taiwan	GenBank

Note: FeLV01–FeLV14 sequences were amplified from plasma viral RNA while the remaining local sequence (FeLVUPM13–FeLVUPM29) were amplified from proviral DNA.

**Table 2:** Predicted transcription binding motifs found within the LTR-gag sequence of Malaysian FeLV isolates.

Motifs	Sequence	Seq length	Coverage	Occurrence in local sequence	Occurrence in reference FeLV
NF-GMa	GAGGTTTCAT	10	523–532	All local seq except UPM08	FeLV-Rickard; FeLV-FAIDs
E1A-F	CGGATGT	7	521–527	UPM08	NA
E1A-F	CGGATGT	7	70–76	UPM18	NA
ELP	CAAGGTC	7	523–527	UPM03, 14, 18, 20	NA
Sp1	GGGGCTAGG	7	521–527	UPM03, 18, 20	NA
C∖EBPbeta	CTGGAAA	7	387–393	UPM18, 20	NA
Krox-20/WT1I-del2	CGCCCCCGC	9	374–382	All local seq	FeLV-Rickard; FeLV-FAIDs
E2F	TTTTGGCGG	9	334–342	UPM03, 14, 18, 20	FeLV-Rickard; FeLV-FAIDs
BAF1	TCCTTGTATACG	12	301–312	All except UPM03, 14, 18, 20	NA
BAF1	TCCTTGTATACG	12	158–169	All local seq	NA
AP-2	CCCAACCG	8	243–250	All local seq	FeLV-Rickard; FeLV-FAIDs
AP-2	CCCAACCG	8	59–66	All local seq except UPM17	FeLV-Rickard; FeLV-FAIDs
AP-2	CCCAACCG	8	58–65	UPM03, 14, 18, 20, 25	NA
AP-2	CCCAACCG	8	4 to 11	All except UPM03, 14	NA
AP-2	CCCAACCG	8	3 to11	All local seq	FeLV-Rickard
GCF	CCGCCCC	7	93–99	All local seq	FeLV-Rickard, FeLV-FAIDs
GCF	CCGGCGC	7	64–70	All local seq except UPM03, UPM 14	NA
GCF	GCGCGCC	7	26–32	UPM27	NA
HNF-3	TGTTTGC	7	129–135	All local seq except UPM06	NA
PEA3	GCGGAAGT	8	69–76	UPM18	NA
TBP	TATAAAA	7	39–45	All local seq	FeLV-Rickard, FeLV-FAIDs
TFIIF-beta	TATAAAA	7	39–45	All local seq	FeLV-Rickard, FeLV-FAIDs
TRF	TATAAAA	7	39–45	All local seq	FeLV-Rickard, FeLV-FAIDs
TFIID	TATAAAA	7	39–45	All local seq	FeLV-Rickard, FeLV-FAIDs
TFIID	CTTCTCGC	8	10 to 17	UPM03, 14, 20	NA
MEP-1	TATAAAA	7	23–29	All local seq except UPM03, 14, 18, 20	FeLV-Rickard
MBF-I	TATAAAA	7	23–29	All local seq except UPM03, 14, 18, 21	FeLV-Rickard
MTF-1	TATAAAA	7	23–29	All except UPM03, 14, 18, 22	FeLV-Rickard

NA: not applicable or not present; seq: sequences.

**Table:** (a)

	FeLV-Rickard [AF052723]	FeLV-FAIDS [M18247]	FeLV-UPM03 [HQ197369]	FeLV-UPM18 [JF815541]	FeLV-UPM20 [JF815543]	FeLV-UPM29 [JF815552]	FeLV-UPM28 [JF815551]	FeLV-UPM12 [HQ727890]	FeLV-UPM06 [HQ197372]
FeLV-Rickard [AF052723]		98.12	94.925	93.609	93.797	96.805	96.805	96.992	96.617
FeLV-FAIDS [M18247]	98.12		95.301	93.985	94.173	96.429	96.429	96.617	96.617
FeLV-UPM03 [HQ197369]	94.925	95.301		96.429	98.496	94.737	94.737	94.925	94.549
FeLV-UPM18 [JF815541]	93.609	93.985	96.429		96.053	93.797	93.797	93.985	93.609
FeLV-UPM20 [JF815543]	93.797	94.173	98.496	96.053		93.609	93.609	93.797	93.421
FeLV-UPM29 [JF815552]	96.805	96.429	94.737	93.797	93.609		100	99.812	99.436
FeLV-UPM28 [JF815551]	96.805	96.429	94.737	93.797	93.609	100		99.812	99.436
FeLV-UPM12 [HQ727890]	96.992	96.617	94.925	93.985	93.797	99.812	99.812		99.624
FeLV-UPM06 [HQ197372]	96.617	96.617	94.549	93.609	93.421	99.436	99.436	99.624	
FeLV-UPM23 [JF815546]	96.805	96.429	94.737	93.797	93.609	99.248	99.248	99.436	99.06
FeLV-UPM21 [JF815544]	96.805	96.429	94.737	93.797	93.609	99.248	99.248	99.436	99.06
FeLV-UPM24 [JF815547]	96.805	96.429	94.737	93.797	93.609	99.248	99.248	99.436	99.06
FeLV-UPM26 [JF815549]	96.805	96.429	94.737	93.797	93.609	99.248	99.248	99.436	99.06
FeLV-UPM09 [HQ197375]	96.805	96.429	94.737	93.797	93.609	99.248	99.248	99.436	99.06
FeLV-UPM17 [JF815540]	96.617	96.241	94.549	93.609	93.421	99.06	99.06	99.248	98.872
FeLV-UPM16 [JF815539]	96.617	96.241	94.549	93.609	93.421	99.06	99.06	99.248	98.872
FeLV-UPM25 [JF815548]	96.429	96.053	94.549	93.797	93.421	98.872	98.872	99.06	98.684
FeLV-UPM19 [JF815542]	95.865	95.489	93.797	92.857	92.669	98.308	98.308	98.496	98.12
FeLV-UPM27 [JF815550]	96.617	95.865	94.173	93.233	93.045	98.684	98.684	98.872	98.496
FeLV-UPM22 [JF815545]	96.617	96.053	94.361	93.421	93.233	98.872	98.872	99.06	98.684
FeLV-UPM10 [HQ197376]	96.429	96.053	94.361	93.421	93.233	99.248	99.248	99.436	99.06
FeLV-UPM15 [JF815538]	96.992	96.617	94.925	93.985	93.797	99.436	99.436	99.624	99.248
FeLV-UPM11 [HQ197377]	96.992	96.617	94.925	93.985	93.797	99.436	99.436	99.624	99.248
FeLV-UPM13 [HQ727891]	96.805	96.429	94.737	93.797	93.609	99.624	99.624	99.812	99.436
FeLV-UPM05 [HQ197371]	96.429	96.053	94.361	93.421	93.233	99.248	99.248	99.436	99.06
FeLV-UPM07 [HQ197373]	96.805	96.429	94.737	93.797	93.609	99.624	99.624	99.812	99.436
FeLV-UPM02 [HQ197368]	96.805	96.429	94.737	93.797	93.609	99.624	99.624	99.812	99.436
FeLV-UPM01 [HQ197367]	96.805	96.429	94.737	93.797	93.609	99.624	99.624	99.812	99.436
FeLV-UPM04 [HQ197370]	96.617	96.241	94.549	93.609	93.421	99.06	99.06	99.248	98.872
FeLV-UPM08 [HQ197374]	94.737	93.985	92.669	91.729	91.541	97.18	97.18	97.368	96.992
FeLV-UPM14 [HQ727892]	95.865	96.241	99.06	96.992	97.932	95.677	95.677	95.865	95.489

**Table:** (b)

	FeLV-UPM23 [JF815546]	FeLV-UPM21 [JF815544]	FeLV-UPM24 [JF815547]	FeLV-UPM26 [JF815549]	FeLV-UPM09 [HQ197375]	FeLV-UPM17 [JF815540]	FeLV-UPM16 [JF815539]	FeLV-UPM25 [JF815548]	FeLV-UPM19 [JF815542]	FeLV-UPM27 [JF815550]
FeLV-Rickard [AF052723]	96.805	96.805	96.805	96.805	96.805	96.617	96.617	96.429	95.865	96.617
FeLV-FAIDS [M18247]	96.429	96.429	96.429	96.429	96.429	96.241	96.241	96.053	95.489	95.865
FeLV-UPM03 [HQ197369]	94.737	94.737	94.737	94.737	94.737	94.549	94.549	94.549	93.797	94.173
FeLV-UPM18 [JF815541]	93.797	93.797	93.797	93.797	93.797	93.609	93.609	93.797	92.857	93.233
FeLV-UPM20 [JF815543]	93.609	93.609	93.609	93.609	93.609	93.421	93.421	93.421	92.669	93.045
FeLV-UPM29 [JF815552]	99.248	99.248	99.248	99.248	99.248	99.06	99.06	98.872	98.308	98.684
FeLV-UPM28 [JF815551]	99.248	99.248	99.248	99.248	99.248	99.06	99.06	98.872	98.308	98.684
FeLV-UPM12 [HQ727890]	99.436	99.436	99.436	99.436	99.436	99.248	99.248	99.06	98.496	98.872
FeLV-UPM06 [HQ197372]	99.06	99.06	99.06	99.06	99.06	98.872	98.872	98.684	98.12	98.496
FeLV-UPM23 [JF815546]		100	100	100	100	99.812	99.812	99.624	99.06	99.436
FeLV-UPM21 [JF815544]	100		100	100	100	99.812	99.812	99.624	99.06	99.436
FeLV-UPM24 [JF815547]	100	100		100	100	99.812	99.812	99.624	99.06	99.436
FeLV-UPM26 [JF815549]	100	100	100		100	99.812	99.812	99.624	99.06	99.436
FeLV-UPM09 [HQ197375]	100	100	100	100		99.812	99.812	99.624	99.06	99.436
FeLV-UPM17 [JF815540]	99.812	99.812	99.812	99.812	99.812		99.624	99.436	98.872	99.248
FeLV-UPM16 [JF815539]	99.812	99.812	99.812	99.812	99.812	99.624		99.436	98.872	99.248
FeLV-UPM25 [JF815548]	99.624	99.624	99.624	99.624	99.624	99.436	99.436		98.684	99.06
FeLV-UPM19 [JF815542]	99.06	99.06	99.06	99.06	99.06	98.872	98.872	98.684		98.496
FeLV-UPM27 [JF815550]	99.436	99.436	99.436	99.436	99.436	99.248	99.248	99.06	98.496	
FeLV-UPM22 [JF815545]	99.624	99.624	99.624	99.624	99.624	99.436	99.436	99.248	98.684	99.248
FeLV-UPM10 [HQ197376]	99.624	99.624	99.624	99.624	99.624	99.436	99.436	99.248	98.684	99.06
FeLV-UPM15 [JF815538]	99.812	99.812	99.812	99.812	99.812	99.624	99.624	99.436	98.872	99.248
FeLV-UPM11 [HQ197377]	99.812	99.812	99.812	99.812	99.812	99.624	99.624	99.436	98.872	99.248
FeLV-UPM13 [HQ727891]	99.624	99.624	99.624	99.624	99.624	99.436	99.436	99.248	98.684	99.06
FeLV-UPM05 [HQ197371]	99.248	99.248	99.248	99.248	99.248	99.06	99.06	98.872	98.308	98.684
FeLV-UPM07 [HQ197373]	99.624	99.624	99.624	99.624	99.624	99.436	99.436	99.248	98.684	99.06
FeLV-UPM02 [HQ197368]	99.624	99.624	99.624	99.624	99.624	99.436	99.436	99.248	98.684	99.06
FeLV-UPM01 [HQ197367]	99.624	99.624	99.624	99.624	99.624	99.436	99.436	99.248	98.684	99.06
FeLV-UPM04 [HQ197370]	99.06	99.06	99.06	99.06	99.06	98.872	98.872	98.684	98.12	98.496
FeLV-UPM08 [HQ197374]	96.805	96.805	96.805	96.805	96.805	96.617	96.617	96.429	96.053	96.617
FeLV-UPM14 [HQ727892]	95.677	95.677	95.677	95.677	95.677	95.489	95.489	95.489	94.737	95.113

**Table:** (c)

	FeLV-UPM22 [JF815545]	FeLV-UPM10 [HQ197376]	FeLV-UPM15 [JF815538]	FeLV-UPM11 [HQ197377]	FeLV-UPM13 [HQ727891]	FeLV-UPM05 [HQ197371]	FeLV-UPM07 [HQ197373]	FeLV-UPM02 [HQ197368]	FeLV-UPM01 [HQ197367]	FeLV-UPM04 [HQ197370]	FeLV-UPM08 [HQ197374]	FeLV-UPM14 [HQ727892]
FeLV-Rickard [AF052723]	96.617	96.429	96.992	96.992	96.805	96.429	96.805	96.805	96.805	96.617	94.737	95.865
FeLV-FAIDS [M18247]	96.053	96.053	96.617	96.617	96.429	96.053	96.429	96.429	96.429	96.241	93.985	96.241
FeLV-UPM03 [HQ197369]	94.361	94.361	94.925	94.925	94.737	94.361	94.737	94.737	94.737	94.549	92.669	99.06
FeLV-UPM18 [JF815541]	93.421	93.421	93.985	93.985	93.797	93.421	93.797	93.797	93.797	93.609	91.729	96.992
FeLV-UPM20 [JF815543]	93.233	93.233	93.797	93.797	93.609	93.233	93.609	93.609	93.609	93.421	91.541	97.932
FeLV-UPM29 [JF815552]	98.872	99.248	99.436	99.436	99.624	99.248	99.624	99.624	99.624	99.06	97.18	95.677
FeLV-UPM28 [JF815551]	98.872	99.248	99.436	99.436	99.624	99.248	99.624	99.624	99.624	99.06	97.18	95.677
FeLV-UPM12 [HQ727890]	99.06	99.436	99.624	99.624	99.812	99.436	99.812	99.812	99.812	99.248	97.368	95.865
FeLV-UPM06 [HQ197372]	98.684	99.06	99.248	99.248	99.436	99.06	99.436	99.436	99.436	98.872	96.992	95.489
FeLV-UPM23 [JF815546]	99.624	99.624	99.812	99.812	99.624	99.248	99.624	99.624	99.624	99.06	96.805	95.677
FeLV-UPM21 [JF815544]	99.624	99.624	99.812	99.812	99.624	99.248	99.624	99.624	99.624	99.06	96.805	95.677
FeLV-UPM24 [JF815547]	99.624	99.624	99.812	99.812	99.624	99.248	99.624	99.624	99.624	99.06	96.805	95.677
FeLV-UPM26 [JF815549]	99.624	99.624	99.812	99.812	99.624	99.248	99.624	99.624	99.624	99.06	96.805	95.677
FeLV-UPM09 [HQ197375]	99.624	99.624	99.812	99.812	99.624	99.248	99.624	99.624	99.624	99.06	96.805	95.677
FeLV-UPM17 [JF815540]	99.436	99.436	99.624	99.624	99.436	99.06	99.436	99.436	99.436	98.872	96.617	95.489
FeLV-UPM16 [JF815539]	99.436	99.436	99.624	99.624	99.436	99.06	99.436	99.436	99.436	98.872	96.617	95.489
FeLV-UPM25 [JF815548]	99.248	99.248	99.436	99.436	99.248	98.872	99.248	99.248	99.248	98.684	96.429	95.489
FeLV-UPM19 [JF815542]	98.684	98.684	98.872	98.872	98.684	98.308	98.684	98.684	98.684	98.12	96.053	94.737
FeLV-UPM27 [JF815550]	99.248	99.06	99.248	99.248	99.06	98.684	99.06	99.06	99.06	98.496	96.617	95.113
FeLV-UPM22 [JF815545]		99.248	99.436	99.436	99.248	98.872	99.248	99.248	99.248	98.684	96.617	95.301
FeLV-UPM10 [HQ197376]	99.248		99.436	99.436	99.624	99.248	99.624	99.624	99.624	99.06	97.18	95.301
FeLV-UPM15 [JF815538]	99.436	99.436		100	99.812	99.436	99.812	99.812	99.812	99.248	96.992	95.865
FeLV-UPM11 [HQ197377]	99.436	99.436	100		99.812	99.436	99.812	99.812	99.812	99.248	96.992	95.865
FeLV-UPM13 [HQ727891]	99.248	99.624	99.812	99.812		99.624	100	100	100	99.436	97.18	95.677
FeLV-UPM05 [HQ197371]	98.872	99.248	99.436	99.436	99.624		99.624	99.624	99.624	99.06	96.805	95.301
FeLV-UPM07 [HQ197373]	99.248	99.624	99.812	99.812	100	99.624		100	100	99.436	97.18	95.677
FeLV-UPM02 [HQ197368]	99.248	99.624	99.812	99.812	100	99.624	100		100	99.436	97.18	95.677
FeLV-UPM01 [HQ197367]	99.248	99.624	99.812	99.812	100	99.624	100	100		99.436	97.18	95.677
FeLV-UPM04 [HQ197370]	98.684	99.06	99.248	99.248	99.436	99.06	99.436	99.436	99.436		96.617	95.489
FeLV-UPM08 [HQ197374]	96.617	97.18	96.992	96.992	97.18	96.805	97.18	97.18	97.18	96.617		93.609
FeLV-UPM14 [HQ727892]	95.301	95.301	95.865	95.865	95.677	95.301	95.677	95.677	95.677	95.489	93.609	
